# Contraindications to Whole-Body Cryostimulation (WBC). A position paper from the WBC Working Group of the International Institute of Refrigeration and the multidisciplinary expert panel

**DOI:** 10.3389/fresc.2025.1567402

**Published:** 2025-04-15

**Authors:** Paolo Capodaglio, Angelo Alito, Benoit Michel Duguè, Romain Bouzigon, Giovanni Lombardi, Elzbieta Dorota Miller, Federica Verme, Giuseppe Modaffari, Paolo Piterà, Ewa Ziemann, Jacopo Maria Fontana

**Affiliations:** ^1^ Research Laboratory in Biomechanics, Rehabilitation and Ergonomics, IRCCS, Istituto Auxologico Italiano, San Giuseppe Hospital, Piancavallo, Italy; ^2^ Department of Biomedical, Surgical and Dental Sciences, University of Milan, Milan, Italy; ^3^ Department of Biomedical, Dental Sciences and Morphological and Functional Images, University of Messina, Messina, Italy; ^4^ Laboratoire Mobilité Vieillissement, Exercice (MOVE), Faculté des Sciences du Sport, Université de Poitiers, Poitiers, France; ^5^ UFR STAPS Besançon, Laboratoire C3S (EA4660), Axe Sport Performance, Université de Franche-Comté, Besançon, France; ^6^ Society Inside the Athletes 3.0, Sport Performance Optimization Complex (COPS25), Besançon, France; ^7^ Laboratory of Experimental Biochemistry and Advanced Diagnostics, IRCCS Ospedale Galeazzi-Sant'Ambrogio, Milano, Italy; ^8^ Department of Athletics, Strength and Conditioning, Poznań University of Physical Education, Poznań, Poland; ^9^ Neurological Rehabilitation Department, Medical University of Lodz, Łódź, Poland; ^10^ Department of Clinical and Biological Sciences, University of Turin, Torino, Italy

**Keywords:** cold therapy, contraindications, cryotherapy, rehabilitation, Whole-Body Cryostimulation

## Abstract

**Background:**

Whole-Body Cryostimulation (WBC) is a treatment that involves short exposures of the entire body to very cold and dry air in specially adapted cryochambers. A growing body of literature suggests the safe application of this technique in medical settings.

**Aim:**

The primary purpose of this study was to generate an international consensus on the updated contraindications for WBC through an interactive process of questionnaire interspersed with controlled feedback from a steering committee.

**Design:**

The study design was based on a systematic review of the literature and Delphi methodology.

**Setting:**

Administration of electronic online questionnaires concerning contraindications to WBC.

**Population:**

A multidisciplinary panel of 48 experts in the fields of rehabilitation, cardiology, neurology, endocrinology, oncology, clinical nutrition or in the clinical application of WBC was invited to participate in this consensus study.

**Methods:**

A systematic search of PubMed, Scopus and Embase databases was carried out to identify possible items for inclusion in a form. A two-round Delphi survey was then conducted according to international guidelines, consisting of an electronic online questionnaire. The experts had to rate their agreement with each item in the questionnaires on a 5-point Likert scale. Expert consensus was assessed.

**Results:**

A total of 28 European experts participated in the Delphi survey. The first round consisted of 59 items, 3 of which were discarded after data analysis. The second round was rearranged according to the previous suggestions of the panellists. All 28 experts completed the two rounds. At the end of the survey, consensus was reached and a final list of temporal and absolute contraindications to WBC was identified.

**Conclusions:**

This process resulted in multidisciplinary expert consensus statements on contraindications to WBC. The European experts agreed on most of the decisions and produced a list of contraindications.

**Clinical rehabilitation impact:**

The results provide a robust evidence framework to help clinicians improve clinical practice and patient safety.

## 1 Introduction

Whole-Body Cryostimulation (WBC) is a treatment that involves short exposures of the entire body (including the head) to very cold and dry air in specially adapted cryochambers (1). It was originally developed for treating symptoms in patients with rheumatic conditions and it has been widely used mostly in sports medicine for muscle injury and recovery after physical exercise and training (2). Over the two last decades, WBC has emerged as an exciting non-pharmacological treatment influencing not only inflammatory events at a cellular and physiological level, but also autonomic, metabolic, and neuroendocrine changes which can result in a wide range of beneficial effects on mental and physical functions (3). On top of its acknowledged effectiveness in relieving symptoms in rheumatic (4) and chronic osteoarticular conditions (5, 6), WBC has been shown to be beneficial also in the treatment and rehabilitation of other diseases like multiple sclerosis (7, 8), fibromyalgia (9, 10), mood disorders (11, 12), deterioration of cognitive functions (13), poor sleep quality (14), obesity (3), pain secondary to central sensitization syndromes (15), phantom limb pain (16), functional neurological disorders (17), tinnitus (18), and post-Covid condition (19). According to such recent literature, indications for WBC have outgrown the traditional ones (i.e., musculoskeletal inflammatory conditions) and a range of conditions have been now safely treated using WBC as complementary therapy (12, 15, 17, 19). Two important institutions had previously expressed concerns regarding WBC safety over the last decade (20, 21). However, their analysis and conclusions were flawed in one critical way: partial-body (body in a cryosauna but head remaining outside, via direct injection of liquid nitrogen mist inside the cabin) and whole-body (full exposure in a cryochamber filled with breathable air) technologies have been amalgamated under the alleged banner «whole-body cryotherapy». A recent scoping review emerged in response to such calls for further investigations on WBC safety and critically analysed each reported case of WBC-induced adverse events using well-established reporting standards (22). WBC is associated with relatively infrequent, and mostly minor and transient adverse effects, and despite its pluri-decennial use and large numbers of WBC sessions, especially in some countries (Poland, Italy, France), only a few adverse events have been reported in the literature to date (22). The reported minor adverse events were cases of cold-induced panniculitis, urticaria, headache, dizziness, reactive hypertension, and long-lasting shivering. Two single cases of transient global amnesia and Moyamoya angiopathy, both with spontaneous improvements, were also reported. Two single more severe cases of intracerebral haemorrhage in a 63-year old woman with a long history of ocular migraine and of aortic dissection in a 56-year-old male with a documented history of hypertension and hypercholesterolemia were described. The first case recovered spontaneously a few weeks later, while the second case required endovascular prosthesis. In both cases, other known risk factors could have accounted for the adverse events: the underlying vascular vulnerability of individuals prone to migraine could have in part accounted for the intracerebral haemorrhage case and the coexistence of arterial hypertension, one of the contraindications to WBC, and overtraining (the patient described himself to be an “avid runner” at the time the event occurred) could have increased the risk factors for aortic dissection (22). The adverse reactions reported could have been prevented with scrupulous medical screening and knowledge of the whole range of contraindications to WBC. The authors of this review (22) concluded that looking back on the past four decades of WBC use, adverse events appear to be rare in relation to the volume of WBC sessions provided worldwide. However, underreporting and a lack of uniform reporting standards may also have accounted for the lack of evidence on adverse events in the literature. Historically, a first list of contraindications to WBC was proposed in a consensus conference that took place at the Second Austrian Symposium on WBC in 2006 in Bad Vöslau (23). A more recent contraindications list was proposed by the Working Group on Whole-Body Cryostimulation of the International Institute of Refrigeration in 2020 based on the evidence emerging from clinical studies published after 2006 (24). The Working Group recommendations were that people with uncontrolled hypertension, chronic migraine, cardiopathies, cold allergy, Raynaud's syndrome, sickle cell anaemia, cryoglobulinemia, claustrophobia, severe hypothyroidism should not be exposed to WBC. Despite those published contraindications, studies on WBC still report non-univocal, general, partial ranges of contraindications or no contraindications at all (see paragraph 3.2 Contraindications). Also, in view of the growing body of clinical studies published after 2020 on the effects of cryogenic cold exposure on conditions not previously treated with WBC, the contraindications list appears now too generic and outdated. Due to the wider use of medically driven WBC, the Working Group on Whole-Body Cryostimulation of the International Institute of Refrigeration (PC, BD, RB, GL, EM, EZ) has felt the need to reformulate the list of contraindications on the basis of a systematic review of the most recent scientific evidence and a Delphi procedure among acknowledged experts. We selected an international multidisciplinary panel of experts, which included: (1) recognised experts in WBC, (2) distinguished European Rehabilitation specialists, (3) prominent specialists in cardiology, neurology, endocrinology, oncology and clinical nutrition. The reasons for such panel composition were that experts using WBC in rehabilitation settings and authoring WBC publications are relatively few, therefore we added in the selection Rehabilitation experts with a scientific role in the European society of Physical and Rehabilitation Medicine with no specific expertise in WBC but mastering the use of physical therapies (among which traditionally used cold therapies). Also, distinguished specialists in cardiology, neurology, endocrinology, oncology and clinical nutrition who disclosed interest about the clinical implications of WBC treatment in their respective fields were invited to join the panel with the aim of providing a specialistic perspective and clinical reasoning about possible specific contraindications to WBC. The primary purpose of this Delphi study was to generate an international consensus on the updated contraindications to WBC through an interactive process of questionnaire interspersed with controlled feedback from a Steering Committee (the Working Group on Whole-Body Cryostimulation of the International Institute of Refrigeration).

## 2 Material and methods

### 2.1 Study design

The study design was based on a systematic review and Delphi methodology with the aim of reaching a consensus on the main contraindications to WBC. The study was conducted in full concordance with the principles of the Declaration of Helsinki and will be reported according to the Conducting and REporting DElphi Studies (CREDES) Guideline (25). The study will be published in a peer-review journal with the authorship agreed as per ICMJE requirements.

### 2.2 Literature review

A systematic review was conducted to identify the most common contraindications reported in the literature and was carried out to answer the review question: “What are the most important contraindications to the use of WBC?”.

### 2.3 Search processing

A systematic review was conducted through a literature search in the databases PubMed, Scopus, and Embase up to 28 February 2023. A specific search strategy was designed for each database to ensure high accuracy and precision. The search method was developed by combining words related to the application WBC. The search strategy is provided in Table 1.

**Table 1:** Search strategy.

Database	String research
Pubmed	(“whole body cryotherapy” OR “whole body cryostimulation”)
Scopus	(“whole body cryotherapy” OR “whole body cryostimulation”)
Embase	“whole body cryotherapy”/exp OR “whole body cryotherapy” OR “whole body cryostimulation”/exp OR “whole body cryostimulation”

### 2.4 Inclusion and exclusion criteria

The criteria for inclusion were (a) original articles, (b) on the use of WBCs. Exclusion criteria were animal or cell studies and articles not available in full text.

### 2.5 Selection of sources

**Figure 1:**
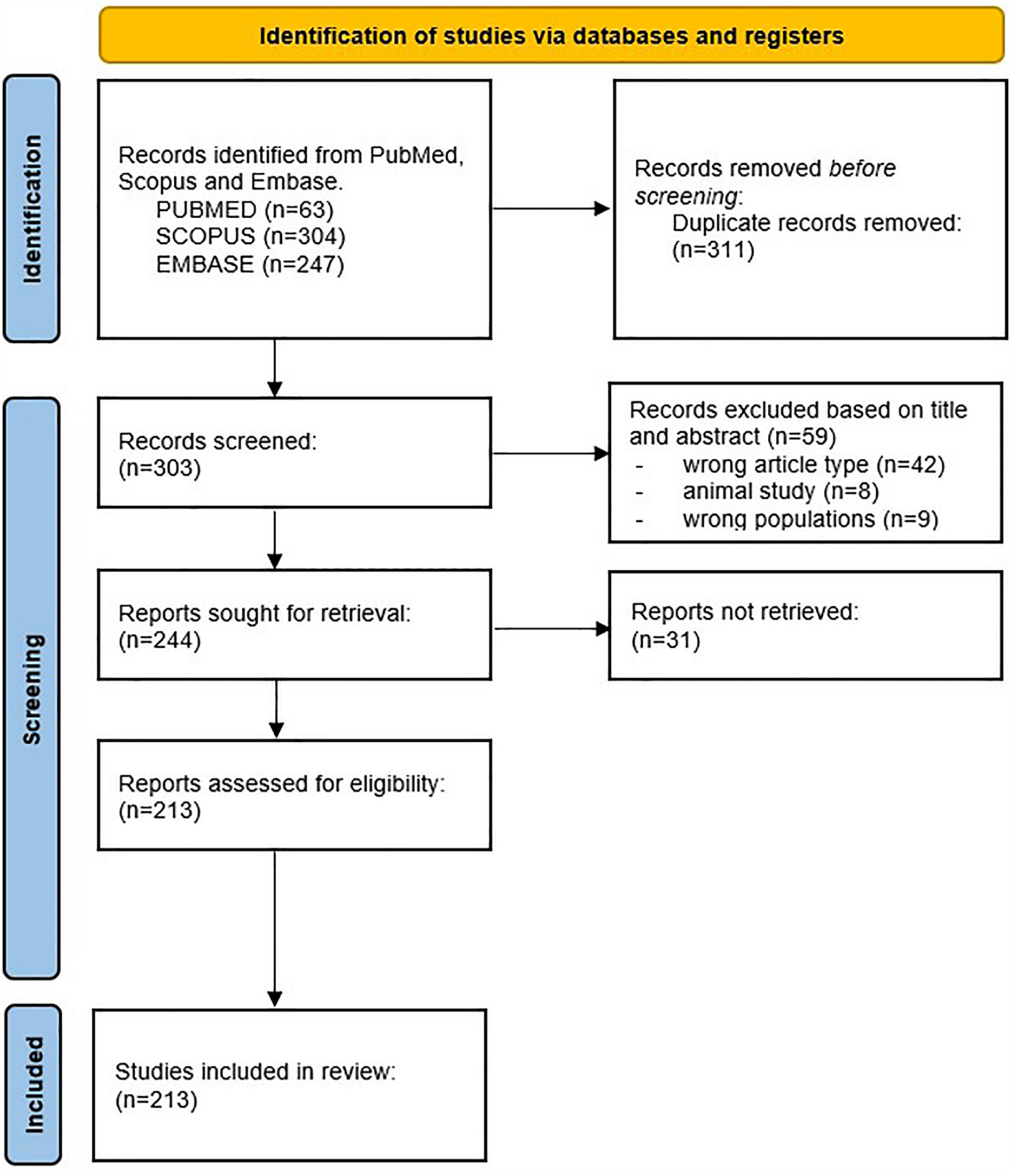
Flowchart describing the selection process of papers, with the preferred reporting items for systematic reviews and meta-analyses (PRISMA).

Four reviewers (AA, GM, FV, PP) searched the database to extrapolate the studies according to the selection criteria, which were then imported into the Rayyan online software (Intelligent Systematic Review) (26) to allow the simultaneous assessment of the studies. The software was then used to detect and remove any duplicate articles. The reviewers independently evaluated the titles and abstracts of the studies according to the eligibility criteria. All articles that met the inclusion criteria were selected for full-text reading. Any disagreements were resolved by consensus. The PRISMA flow chart (27) is shown in Figure 1.

### 2.6 Data extraction

Data extraction was then completed independently by other two authors (JMF, and PC), who also contributed to the analysis process. The accuracy of the data was confirmed by a third author (AA). Disagreements were resolved by consensus. Relevant data were then entered into a database on (1) first author and year, (2) contraindication sorted by temporary or absolute contraindication or system organ, (3) absence of contraindication, (4) reference to another study for contraindication. Based on the number of comparisons in the selected studies, the data were then summarised.

### 2.7 Delphi procedure

The Delphi method consists of the search for a common consensus among a group of experts on a specific topic (28, 29). It is a well-known method for determining a consensus opinion among subject matter experts to respond to a research question. Participants are allowed to nuance and reevaluate their views depending on the anonymous perspectives of others, allowing for participant evaluation. The structured Delphi process for reaching consensus among panellists has been widely accepted in a variety of medical specialties. In this case, the Delphi study was conducted based on a systematic review and then through consecutive rounds of questionnaires. Anonymity of panellists in the survey rounds, controlled feedback and iterative discussions were the strengths of this Delphi procedure.

The first-round questionnaire was prepared by the Steering Committee and included all rating items obtained from the literature search previously conducted through the systematic review. After discussion within the multidisciplinary Steering Committee and with the specialists in the Panel of Experts, the authors decided to break down items that were too general and insufficiently precise into more specific sub-items. A Google Form questionnaire was then prepared for a first Delphi round in which panellists were asked to express their level of agreement on a 5-point Likert scale with all the medical contraindications reported and, finally, to comment on their wording and comprehensiveness.

### 2.8 Definition of consensus

The authors decided that the response rate of the first Delphi round should not fall below 70% (30). Those items that scored 3, 4 or 5 points in the first round in 70% or more of the cases were directly included in the second-round rating form. Items that scored 1 or 2 points in 70% of the cases were discarded directly from the form. Instability of the responses after the first round would lead to a second round reformulated by the Steering Committee. Only in case of stability of the responses after the first round of Delphi, the items would have been evaluated in the second round through dichotomous (Yes/No) questions about the need to include each item in the contraindications' list. Items that scored “Yes” in 80% or more of the cases were included in the final contraindications' list.

### 2.9 Expert panel

A total of 48 experts were invited to participate in this study through a purposive and snowball sampling between 1 February and 16 March 2024. The inclusion criteria for participation in the study were (a) being an acknowledged expert user of WBC who authored peer-reviewed WBC studies, (b) being a distinguished Rehabilitation specialist with a scientific role in the European Society of Physical and Rehabilitation Medicine or (c) being a specialist in cardiology, neurology, endocrinology, oncology, clinical nutrition with an interest in the clinical applications of WBC.

## 3 Results of the systematic review

### 3.1 Study selection

A total of 614 potentially relevant papers were identified by searching the PubMed, Scopus and Embase databases. 311 duplicates were excluded from the search before screening. After reading the title and abstract, 59 articles were discarded for the following reasons: wrong article type (i.e., case report, case series, review) (*n* = 42), animal study (*n* = 8), wrong population study (*n* = 9). Of the remaining 244 articles, a total of 31 studies could not be retrieved. The full text of the remaining 213 articles was read and all were suitable for data extraction (Figure 1). An overview of the included studies and the extracted data is given in Supplementary Material.

### 3.2 Contraindications

Seventy-four studies cited no contraindications in the manuscript. Sixty-nine articles cited only general contraindications without specifying the reference. Thirty-three studies cited referenced articles, in particular: 10 studies cited Lubkowska et al. 2012 (31), 9 studies cited Gregorowicz and Zagrobelny (32), 4 studies cited Lombardi, et al. (33), 3 studies cited Lubkowska et al. 2015 (34), 2 studies cited Sieron, et al. (35), 2 studies cited Dugué, et al. (24), 1 study cited Księżopolska-Pietrzak (36), 1 study cited Legrand, et al. (22), 1 study cited Rymaszewska et al. (37).

From this review, 59 contraindications to WBC reported in the literature were found. The multidisciplinary Steering Committee classified them into different categories, as shown in Table 2.

**Table 2:** A comprehensive review of contraindications to WBC reported in the literature reviewed (up to 28 February 2023).

Absolute contraindications due to transitory conditions		
Current infections	Anaemia (severe)	Abrasion Injuries
Blood pressure	Dehydration	Open wounds
Fever	Pregnancy	Emaciation/cachexia
Drug abuse	Addictions	Unable to maintain standing position
Hypothermia	Alcohol abuse	
Subjective absolute contraindications		
Cold intolerance	Cold sensitivity	Claustrophobia
Cardiological diseases		
(Unstable) coronary artery disease	Hypertension (uncontrolled/unstable/untreated)	Myocardial infarction (acute or recent)
(Unstable) Angina pectoris	(History of) Arrhythmias (acute or severe)	Acute cardiovascular diseases
Valvopathies (stenosis, insufficiency)	Pacemaker	
Vascular conditions		
(Recent history or acute) venous thromboembolic events	Peripheral arterial disease (stage 3–4)	Local blood flow disorders (ischemia, stasis, thrombosis, embolism)
Metabolic/endocrine disorders		
Hypothyroidism	Uncontrolled Diabetes	
Rheumatological/Immunological diseases		
Any form of Active Vasculitis	Raynaud's syndrome	Clotting diseases
Cryoglobulinemia		
Respiratory conditions		
(Acute) respiratory infection/disease	Bronchospasm (cold induced)	Chronic respiratory insufficiency
Asthma (cold induced)	(Symptomatic) lung disease	Chronic obstructive pulmonary disease
Neurological diseases		
Disorder of autonomic/sympathetic nervous system	(Acute or recent) Stroke	Epilepsy
Polyneuropathies	Cognitive disturbances	
Psychiatric diseases		
Psychosis	Mental disorder	Anxiety
Renal/urinary diseases		
Acute and chronic renal diseases	Urinary infections	
Neoplastic diseases		
Active cancer		
Dermatological diseases		
Ulcers	Purulent/gangrenous skin changes	Hyperhidrosis

### 3.3 Participation results

Of the 48 panellists invited to participate in the study, 28 participants from Finland, Israel, Italy, France, Poland, Slovenia, United Kingdom, Turkey agreed to complete Round I of the Delphi study and all of them completed then Round II.

#### 3.3.1 Round I

Of the 59 items proposed to the panellists, 3 items (“Raynaud”, “hyperhidrosis”, “anxiety”) were discarded after reaching low consensus in Round I. The other items 56 items (94%) scored 3, 4 or 5 points in over 70% of the cases and were included by the Steering Committee in the Round II questionnaire. The addition of the item “chronic migraine” was proposed by the Steering Committee based on the paper published in 2023 by Legrand et al (22) and in line with the documented association with increased risk of haemorrhagic stroke. Also the item “age over 80 years” was added based on the panellists' physiological reasoning about the known progressive decrease in thermal perception in the elderly (38).

#### 3.3.2 Round II and final list of contraindications

**Figure 2:**
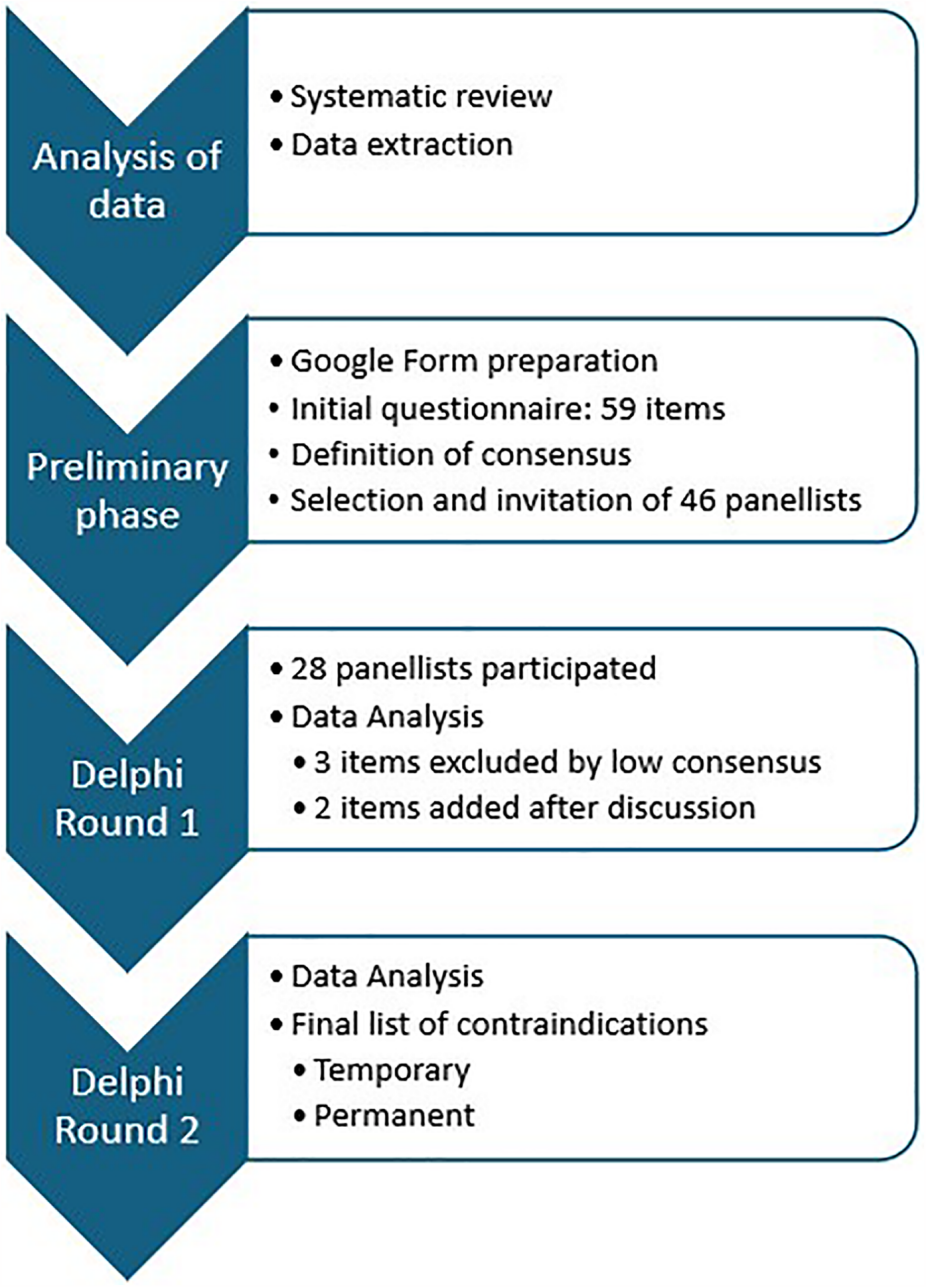
Flowchart illustrating the process of the study and the two Delphi rounds.

The items listed in Round II obtained a consensus rate over 80%. The panellists raised the issue of whether WBC is suitable for subjects under 18 years of age and its eventual inclusion in the contraindications list was discussed. No published data but only unpublished anecdotal reporting (from Paris Olympic games in 2024) about the absence of contraindication to WBC in adolescents exist. After discussion about possible WBC-induced harmful consenquences in subjects under 18 years of age, the Steering Committee decided not to include it in the final contraindications list. A thorough discussion about the oncological items took place with the oncologists in the Panel. As a result, the Steering Committee decided to replace the general item “melanoma” formerly proposed in the literature and include more specific items like “malignant melanoma” and “other aggressive cutaneous cancer irrespectively of treatment (previous or actual)”. On the same line, the item “active cancer” was replaced by the specific items “any untreated, uncontrolled or progressive invasive cancer irrespectively of involved sites” and “any ongoing antineoplastic treatment”. The Steering Committee then came to the definition of the final list of contraindications, classifying them into temporary (i.e., conditions present at the time of medical assessment) and permanent. This classification of contraindications is meant for clinicians, with the aim of facilitating patient assessment and decision-making regarding WBC suitability. A flowchart representing the study process is provided in Figure 2.

***Temporary:*** acute illnesses or infections, blood pressure ≥160/100 mmHg or <100/60 mmHg with hemodynamic instability signs, fever, skin lesions, severe anaemia (Hb ≤ 80 g/L), electrolyte abnormalities, moderate-severe thrombocytopenia (PLT < 50 × 10^9^/L), pregnancy, cachexia, current therapy with cardiotoxic or spasmogenic drugs, any ongoing antineoplastic treatment, excessive skin sweating, general malaise (e.g., dizziness, nausea, weakness, shivering), claustrophobia, cold intolerance.

***Permanent:***

***General:*** age over 80 years, lack of informed consent of the patient.

***Cardiovascular:*** ischaemic heart disease, haemodynamic instability, current or symptomatic myocarditis/pericarditis/endocarditis, uncontrolled hypertension, moderate to severe stenotic valvular heart disease documented by echocardiography, decompensated heart failure, hypertrophic cardiomyopathy and any other forms of outflow tract obstruction, uncontrolled tachyarrhythmias or heart rate >110 bpm, second or third degree atrioventricular block, pacemaker, automatic implantable cardiac defibrillator, peripheral artery diseases (except stage I Fontaine) (39), aortic root or ascending aorta dilatation ≥45 mm, vasculitis, acute/recent thrombophlebitis, phlebothrombosis, pulmonary embolism (<6 months), chronic migraine.

***Endocrinological:*** Uncontrolled thyroid diseases, hypoadrenalism, Type 1 Diabetes, uncontrolled Type 2 Diabetes or complicated by vasculopathy, retinopathy.

***Immunological****:* cold-related immunological diseases (cryoglobulinemia, agglutinins, cryofibrinogenemia, paroxysmal haemoglobinuria, agammaglobulinemia), thrombocytopenia (grade 3–4).

***Organ insufficiency****:* acute and chronic renal insufficiency (Stage III–IV), acute respiratory infection/disease, severe asthma, severe chronic respiratory insufficiency, COPD (stage III–IV), active pulmonary tuberculosis.

***Oncological:*** malignant melanoma or other aggressive cutaneous cancer irrespectively of treatment (previous or actual), any untreated, uncontrolled, or progressive invasive cancer irrespectively of involved sites, any ongoing antineoplastic treatment.

***Neurological/Psychiatric:*** neurovegetative dysautonomia, neuropathies of the sympathetic nervous system, chronic migraine, polyneuropathies, recent stroke (<12 months), epilepsy/seizures, mental conditions excluding the possibility of establishing logical contact, expressing informed consent and adapting to the rules, and engaging in risky behaviour, dementia, exacerbation of psychotic or affective disorders, suicidal tendency, addiction to alcohol and other psychoactive substances.

***Dermatological:*** purulent/gangrenous skin changes.

***Ophthalmological:*** glaucoma.

## 4 Discussion

In consideration of the increasing use of WBCs not only in sports and wellness facilities but also in clinical settings, this position paper from the WBC Working Group of the International Institute of Refrigeration addresses the need to update the list of contraindications based on current scientific evidence. A previous review concluded that WBC is a safe procedure provided it is preceded by accurate medical screening to exclude contraindications and appropriate exposure parameters are prescribed (22). Medical examinations, including blood pressure and skin temperature measurements, are mandatory for persons to be exposed to WBC (22). Historically, WBC studies have been directed towards a limited range of medical conditions, particularly musculoskeletal and rheumatological, and mostly in outpatient populations (4, 6, 9). More recently, WBC studies have been performed in clinical settings on inpatients affected by conditions not previously investigated in the cryochamber, for whom clinical hypotheses of possible beneficial effects had been formulated by clinicians (7, 15, 19). However, the reference contraindications to WBC have remained those proposed in 2020 (24), mostly based on precautionary criteria rather than on the evidence of presence/absence of adverse events in a specific population. Consequently, they were intended to cover a wide range of conditions or stages of specific diseases, in line with the “*primum non nocere”* (first, do no harm) principle of our ancient Latin predecessors. In oncological conditions, little is known about the mechanisms responsible for the biological effects of low temperatures on malignant tumours in humans. Concerns about the potential WBC-induced increased metabolism that might indirectly fuel the diffusion of cancer cells exist but are controversial. Whereas some studies seem to suggest that low temperatures might represent a risk factor for several types of cancer (40–42), others claim opposite evidence (43). According to the latter, cold exposure stimulates the sympathetic nervous system and the non-shivering thermogenesis with brown adipose tissue activation mediated by the uncoupling protein 1, which is essential for cold-induced tumour suppression (43).

From a clinical point of view, the studies conducted to determine the effects on neoplastic cells activation or spread have been performed on tissues, and there is no evidence to date that the application setting of WBC recreates a biological environment suitable for the activation and spread of neoplastic cells in the human body. However, in the absence of specific evidence, patients' safety should remain a primary concern. WBC users should be aware that the scientific evidence about the use of WBC in neoplastic conditions is non-existing at all. Generally, a precautionary principle about enrolling oncological patients into WBC protocols should prevail and an oncological opinion requested before prescribing WBC in those patients.

Specifically, the oncologists in the panel, based on the potentially WBC-induced increased metabolism, indicated to include in the final contraindications list the items “malignant melanoma”, “aggressive cutaneous cancer irrespectively of treatment (previous or actual)”, “untreated, uncontrolled, or progressive invasive cancer irrespectively of involved sites”, as well as “any ongoing antineoplastic treatment”.

In cardiological conditions, the scientific evidence about the use of WBC is unpublished and anecdotal. After discussion with the cardiologists in the panel, the Steering Committee's opinion is that the widely used clinical contraindications to ergometric testing can be relied upon, since the sympathetic load of an ergometric test can be assimilated to that induced by WBC.

In general, the evidence on the use of WBC in conditions other than rheumatological and musculoskeletal remains low and nascent because of lack of studies due to the traditional concern in applying cold-induced stress to potentially risky conditions, the small sample sizes of the existing studies, sometimes just case-reports, the few controlled randomized trials and the low quality of published studies.

For the time being, authors recommend a use of WBC in sports-related muscle recovery, in spas for general well-being and in clinical setting for selected clinical conditions, preceded by targeted medical screening. In addition to that, clinical reasoning about possible negative synergies of risk factors, not currently listed as single contraindications *per se,* should also be conducted. As already pointed out by Legrand et al. (22), the potential risks linked to the combination of different, moderate or even asymptomatic conditions (i.e., lipid disorders and aortic aneurysm), should also be taken into account. It is mandatory that WBC prescription for clinical conditions is medically driven and the appropriate setting is a clinical one. The main scope of this paper was to update the list of contraindications to WBC based on the latest studies to serve as a practical clinical guidance for recommended screening procedures for prescribing WBC in clinical conditions. In the wake of this contraindications list, decision-making algorithms for routine clinical workflows could now be implemented. As new research on WBC applications becomes available up-to-date summaries will be needed. Issues such as accreditation of facilities and curricula for WBC users will also need to be addressed. Ongoing and future studies should provide higher quality evidence and eventually lead to a wider use of medically driven WBC in rehabilitation with a reformulation of the medical indications and contraindications for WBC.

This study has some limitations. Delphi designs have a known limitation of potential researcher bias in the formulation of preliminary statements. To overcome this concern, the initial form was developed solely from a systematic review of the international literature. We used a general search string, not only on contraindications to WBC, because literature on the topic is still scarce, including chapters of books or papers not written in English. The 70% agreement rate in round 1 is lower compared to some other Delphi studies but in line with published methods (44). On the other hand, round 1 is traditionally used mostly to generate ideas and comments among the panel members about the contraindications published in the available literature (44). Using a higher consensus cut-off of 80% agreement in Round 2 was a decision based upon the need to reach a strong consensus on those items considered core for the final contraindications list. It is known that the iterative nature of the Delphi method can lead to biases like dominance and group conformity (defined as groupthink), which may suppress divergent views and result in an excessively homogeneous consensus opinion that overlooks important contraindications or risks associated with WBC. Experts' personal biases, experiences and perspectives may influence their responses throughout the Delphi process, affecting the consensus reached. The anonymity of individual members in our Delphi study removes the inherent groupthink bias observed with face-to-face group meetings. Also, being the identification and selection of panel members discrepant in published Delphi studies, we decided to select a heterogeneous panel to achieve a broader perspective and generalization of consensus. Appropriate size of the panel depends on the complexity of the problem, homogeneity or heterogeneity of the panel, and availability of the resources. Generally, a number close to 30–50 is considered optimum in concluding rounds for a homogenous Delphi (45). With that in mind, and aware of the limited competent resources that could address our specific topic, we invited 48 experts among acknowledged users of WBC who authored peer-reviewed WBC studies, specialists in Rehabilitation, cardiology, neurology, endocrinology, oncology, clinical nutrition with an interest in the clinical applications of WBC in their respective fields. Only 28 experts, in line with our selection criteria and representing all the competences required to address the Delphi Rounds' questions, replied to the invitation. We did not observe a decline in response rate between the two rounds and experts who had agreed to participate stayed involved until the process was completed. The panel size (close to 30), the diverse representation from different specialties and geographical distribution of members can be considered appropriate for the generalizability of this Delphi results.

## 5 Conclusions

The present Position Paper of the WBC Working Group of the International Institute of Refrigeration and a multidisciplinary Panel of international experts attempts to fill the gap between the acknowledged contraindications and the growing scientific evidence and provide updated recommendations on the contraindications to WBC. This process resulted in expert consensus statements on contraindications to WBC. The experts agreed on most decisions and produced a list of contraindications. The results provide a robust framework to help clinicians improve clinical practice and patient safety.

Accurate medical assessment, critical appraisal and screening remain mandatory before WBC prescription. In prescribing WBC, appropriate exposure parameters, in terms of session length or chamber temperature, should be observed to avoid risks (22). The contraindications hereby proposed are meant to guide the initial medical screening. However, the decision to include a patient in WBC treatment should be based on a careful and comprehensive medical assessment of the presence of combined subclinical risk factors. Therefore, medical warnings about possible combined risk factors should be present and WBC prescription should be guided by precautionary criteria about possible association of risk factors not necessarily included in the list of contraindications. Temporary contraindications, such as general malaise, can be postponed until the patient has recovered. For subjective contraindications such as claustrophobia and sensitivity to cold, the clinician should use clinical judgement to assess whether the WBC session can be performed after medical suasion and under supervision.

## Group members of expert panel members

Helena BURGER (rehabilitation), Amelia BRUNANI (endocrinology), Raffaella CANCELLO (nutrition), Roberto CASALE (neurophysiology), Maria Gabriella CERAVOLO (neurology), Marco CORDERO (cardiology), Joseph T. COSTELLO (WBC expert), Massimo DE NARDI (WBC expert), Olivier DUPUY (WBC expert), Paolo FANARI (pneumology), Simmie FOSTER (psychiatry), Klemen GRABLJEVEC (rehabilitation), Claudio GRAIFF (oncology), Fabien LEGRAND (WBC expert), Julien LOUIS (WBC expert), Anna LUBKOWSKA (WBC expert), Neža MAJDIČ (rehabilitation), Aydan ORAL (rehabilitation), Juha OKSA (WBC expert), Alessandro PICELLI (rehabilitation), Rachele POJEDNIC (nutrition), Guillaume POLIDORI (WBC expert), Nicola POLVERELLI (oncology), Joanna RYMASZEWSKA (WBC expert), Isabella SPRINGHETTI (oncological rehabilitation), Agata STANEK (WBC expert), Piotr TEDERKO (rehabilitation), Iuly TREGER (rehabilitation).
